# Autosomal recessive A20 zinc finger 7 mutation is associated with early-onset lupus-like disease

**DOI:** 10.1007/s00011-026-02326-2

**Published:** 2026-07-18

**Authors:** Mohamed Alsabbagh, Satanay Z. Hubrack, Lara Gamgoum, Maryiam J. A. Osman, Katherine E. Ford, Alya Al Shakaki, Amal Robay, Housam Sarakbi, Samar Al Emadi, Rafah Mackeh, Khalid A. Fakhro, Bernice Lo

**Affiliations:** 1 Translational Medicine, Sidra Medicine, Doha, Qatar; 2 College of Health and Life Sciences, Hamad Bin Khalifa University (HBKU), Doha, Qatar; 3 Department of Genetic Medicine, Weill Cornell Medicine – Qatar (WCM-Q), Doha, Qatar; 4 Rheumatology Division, Hamad Medical Corporation, Doha, Qatar; 5 Present Address: University of Louisville, Kentucky, Louisville, KY USA

**Keywords:** A20, Impaired NF-κB, Linear ubiquitin, Zinc finger 7, Lupus

## Abstract

**Background:**

A20 is an anti-inflammatory protein that suppresses nuclear factor-κB (NF-κB)-mediated inflammatory gene expression and inhibits cell death. Disruption of A20 function results in defective suppression of the NF-κB pathway, manifesting in diverse autoimmune and autoinflammatory conditions. While autosomal dominant A20 mutations have been identified to cause autoinflammatory disease, recessive A20 mutations causing disease have not been previously described.

**Methods:**

We utilized whole exome sequencing to identify the variant of interest. In silico structural modeling as well as immunoprecipitation were used to ascertain A20’s interaction with linear ubiquitin, coupled with flow cytometric analysis and western blotting to measure the expression of NF-κB activation markers. We quantified NF-κB pathway activity using NF-κB reporter assay.

**Results:**

Here, we report a novel homozygous mutation in the A20 protein responsible for early-onset lupus-like disease starting at four years of age. We show that the A20 E781K variant, situated in the seventh zinc finger domain of A20, compromises the protein’s ability to bind linear ubiquitin, resulting in a functional hypomorph with a reduced capacity to inhibit NF-κB activation and downstream gene expression in HEK293 cells.

**Conclusion:**

Our findings characterize a critical mutation associated with early-onset lupus-like disease in its recessive form and continue to highlight the important role of A20 in maintaining immune homeostasis.

**Supplementary Information:**

The online version contains supplementary material available at 10.1007/s00011-026-02326-2.

## Introduction

The transcription factor nuclear factor kappa B (NF-κB) is a master regulator of immune homeostasis, orchestrating processes essential for both innate and adaptive immunity, inflammation, and cell survival. The NF-κB pathway integrates signals from several pathways, including those mediated by tumor necrosis factor receptor 1 (TNFR1), to coordinate gene transcription in response to diverse internal and external stimuli.

Activation of the NF-κB pathway is initiated upon the engagement of proinflammatory cytokines, such as tumor necrosis factor (TNF), with its respective receptor TNFR1. This triggers the formation of the TNFR1 signaling complex (TNF-RSC), which in turn recruits and activates the IKK complex, consisting of IKKα, IKKβ, and the NF-κB essential modulator (NEMO). Activation of the IKK complex leads to the degradation of the inhibitory molecule IκBα, resulting in the phosphorylation and subsequent translocation of the NF-κB heterodimer to the nucleus, where it can initiate the transcription of its downstream target genes to mount a proper immune response [1].

Given its central role, the NF-κB pathway is tightly regulated by several mechanisms to prevent excessive inflammation, cell death, or tissue damage. One key regulatory mechanism is polyubiquitination, a post-translational modification that assists in signal transduction and protein scaffolding. Ubiquitin chains form when ubiquitin molecules attach via residue G76 to lysine residues or the N-terminal methionine residue of another ubiquitin molecule, with the type of linkage determining the functional outcome. For instance, K63-linked ubiquitination promotes the recruitment of downstream molecules in signaling pathways, while K48-linked ubiquitination targets proteins for proteasomal degradation [2]. Linear polyubiquitin chains (M1-linked), generated by the linear ubiquitin chain assembly complex (LUBAC), play a crucial role in NF-κB activation [3]. These chains are attached to RIPK1 and NEMO, enhancing protein scaffolding, stabilizing the TNF-RSC, allowing for the recruitment of the IKK complex and subsequent NF-κB activation [4, 5].

A key regulator of the NF-κB pathway is the protein A20, encoded by the NF-κB downstream target gene *TNFAIP3*. A20 is a potent inhibitor of the NF-κB pathway acting in a negative feedback loop in response to NF-κB activation [6]. It is comprised of an ovarian tumor (OTU) domain and seven zinc finger (ZF) domains, several of which engage in distinct interactions with components of the NF-κB pathway. The OTU domain has deubiquitinase activity that removes stabilizing K63-linked polyubiquitin from RIPK1 and NEMO [7, 8]. ZF4 was shown to contain E3 ubiquitin ligase activity that attaches K48-linked polyubiquitin, targeting the TNF-RSC member RIPK1 for proteasomal degradation [7]. ZF7, on the other hand, was shown to be required for the recruitment of A20 to the TNF-RSC [9, 10]. Moreover, A20 disrupts NF-κB activation through a nonenzymatic mechanism by binding to linear polyubiquitin chains involved in IKK complex activation via its ZF7 domain [9–11]. In addition to its function as a negative regulator of NF-κB, the ZF7 domain of A20 also prevents cell death by binding linear polyubiquitin chains within TNF-RSC, contributing to the retention of RIPK1 in complex I and thereby preventing its release to form the apoptosis-inducing complex II [12, 13]. Aside from its role in TNFR1 signaling, A20 has been implicated in dampening NF-κB activation downstream of the T and B-cell-receptors, interleukin-1 and 6 receptors, Toll-like receptors, and NOD-like receptors [1, 8, 14, 15].

Loss-of-function mutations in A20 fail to suppress the NF-κB pathway and result in widespread immune dysregulation, which can manifest as autoimmune and autoinflammatory disorders. Multiple genome-wide association studies initially determined *TNFAIP3* to be a susceptibility locus for autoimmune disorders such as systemic lupus erythematosus (SLE) and rheumatoid arthritis [16, 17]. Moreover, reduced A20 mRNA expression was shown to correlate with SLE disease severity [18]. Haploinsufficiency of A20 (HA20), a monogenic disorder caused by heterozygous loss-of-function mutations in A20, is associated with a heterogeneous spectrum of autoimmune and autoinflammatory manifestations, including mucosal ulcers, fever, gastrointestinal issues, thyroiditis, lupus, along with joint involvement [19, 20]. In mouse models, inactivation of A20 results in perinatal lethality due to widespread inflammation, while specific inactivation of ZF7 results in an autoinflammatory phenotype marked by psoriatic-like arthritis and multiorgan inflammation resulting from aberrant MyD88 signaling [21–24]. While A20 and its individual domains have been extensively studied, there is little literature describing the functional contribution of its ZF7 domain to the manifestation of human pathology.

Here, we report a novel homozygous loss-of-function missense mutation in the ZF7 domain of A20 identified in a patient with an early-onset lupus-like disorder. The mutation disrupts A20’s ability to bind linear polyubiquitin, leading to heightened NF-κB activation in HEK293 cells. This case provides further insight into the molecular underpinnings of A20 pathologies and the development of monogenic lupus, as well as highlights the importance of the A20 ZF7 domain in maintaining immune homeostasis.

## Materials and methods

### Genetic analysis of the whole exome sequencing

Whole exome sequencing was performed as previously described [25]. Variants were prioritized based on minor allele frequency (MAF *≤* 0.001 in Genome Aggregation Database, the Exome Aggregation Consortium, and the 1000 Genomes Project), consistency with expected mode of inheritance, in silico predictions of pathogenicity (SIFT, PolyPhen-2, MutationTaster, CADD), and clinical relevance including gene function and phenotype correlation. Only variants affecting protein sequence–nonsynonymous, nonsense, splice-site, or indels–were considered.

### Multiple sequence alignment

The amino acid sequence of human A20 ZF7 (NP_001257436.1) was aligned to the canonical A20 sequences across different species using ClustalW. Alignment was performed in UGENE v40.1.

### In silico prediction of the mutation’s impact on protein structure

The determined crystal structure of A20 ZF7-linear diubiquitin complex was obtained from Protein Data Bank (Entry ID: 3VUW) and used to introduce the E781K mutation via DynaMut2 to determine the complex’s stability. The structures were then visualized and imaged in PyMOL. Pathogenicity of the mutation was estimated via AlphaFold’s AlphaMissense.

### Plasmids and antibodies

pEGFP-C1-A20 was a gift from Yihong Ye (Addgene plasmid #22141; http://n2t.net/addgene:22141 ; RRID: Addgene_22141) and is a plasmid coding for A20 wild-type (WT) [26]. mRFP tagged A20 construct was generated by excising the A20 cDNA from pCMV6-TNFAIP3-Myc-DDK (Origene, RC221337) using the restriction enzymes EcoRI (New England Biolabs #R0101) and NotI (New England Biolabs #R0189) and ligating it into pcDNA3-mRFP, which was a gift from Doug Golenbock (Addgene plasmid #13032; http://n2t.net/addgene:13032; RRID: Addgene_13032) to generate pcDNA3-mRFP-A20. pmaxGFP Vector (Lonza) or pcDNA3-mRFP were used as empty vector controls. pGreenFire1-NF-κB (EF1α-puro) lentivector plasmid (System Biosciences #TR012PA-P), and a pLenti-P2A and pLenti-P2B mixture (abm #LV003) was used to generate the HEK293 NF-κB-GFP stable cell line. The following antibodies were used: linear ubiquitin (Millipore #MABS451), IκBα (Cell Signaling #4814), phospho-NF-κB p65 (Cell Signaling #3033), GFP (Santa Cruz sc-9996), α-Tubulin (Cell Signaling #3873) and phospho-NF-κB p65-PE (Cell Signaling #5733).

### Site-directed mutagenesis and Sanger sequencing

The QuickChange Lightning Site-Directed Mutagenesis Kit (Agilent #210518) was used to introduce c.2341G > A, c.2343A > C point mutations in pEGFP-C1-A20 (Addgene #22141) and pcDNA3-A20-RFP to generate pEGFP-C1-A20-E781K, pcDNA3-mRFP-A20-E781K, pEGFP-C1-A20-E781D, pcDNA3-A20-E781D respectively. Mutations c.2274delC and c.680T > A were also introduced in pEGFP-C1-A20 to generate pEGFP-C1-A20-K759Sfs*56 and pEGPF-C1-A20-L227*, respectively. The mutagenesis was carried out as per the manufacturer’s protocol. The primer pairs used to introduce each mutation are as follows: p.E781K—5′-GCAACGGCTACTGCAACAAATGCTTTCAGTTCAAG-3′, 5′-CTTGAACTGAAAGCATTTGTTGCAGTAGCCGTTGC-3′; p.E781D—5′-GCAACGGCTACTGCAACGACTGCTTTCAGTTCAA-3′, 5′-TTGAACTGAAAGCAGTCGTTGCAGTAGCCGTTGC-3′; c.2274delC—5′-CCGAAGACCCCCCAAGCAGCGTTGCC-3′, 5′-GGCAACGCTGCTTGGGGGGTCTTCGG-3′; c.680T > A—5′-TAAATTCCACCCACTTTCTAAGGGGCGAAATTGGAAC-3′, 5′-GTTCCAATTTCGCCCCTTAGAAAGTGGGTGGAATTTA-3′. Sanger sequencing was used to sequence the entire A20 insert to confirm the desired mutation within A20. The sequences were analyzed by UGENE v40.1 [27].

### Cell culture and transfection

HEK293 or HEK293T cells were cultured in Dulbecco’s Modified Eagle Medium (DMEM) (Cytiva #SH30243.01) containing 10% fetal bovine serum (Thermo Fisher Scientific #10500064), 100 units/mL Penicillin and 100 µg/mL Streptomycin (Thermo Fisher Scientific #15140122) and incubated at 37 °C in 5% CO2. Transient transfections were performed with the indicated vectors using TurboFect Transfection Reagent (Thermo Fisher Scientific #R0531) as per the manufacturer’s instructions and incubated at 37 °C for 24 h.

### Generating HEK293 NF-κB-GFP stable cell line

The HEK293-NF-κB-GFP stable cell line was generated via lentiviral transfection. The following steps were done using serum-free antibiotic-free DMEM unless indicated otherwise. The DNA complex consisting of 5 µg of each pLenti-P2A and pLenti-P2B, and 10 µg pGreenFire1-NF-κB (EF1α-puro) lentivector plasmid in 1 mL DMEM was added to 80 µL of Lipofectamine 2000 Transfection Reagent (Invitrogen #11668019) in 1 mL DMEM. Both the DNA complex and diluted lipofectamine were each incubated at room temperature for 5 min. Once mixed, the solution was then incubated at 37 °C for 20 min. The resulting transfection complex was supplemented with 4 mL FBS and added to 80% confluent HEK293T cells in a 10 cm dish containing 5 mL fresh DMEM and incubated at 37 °C for 5 h. 650 µL FBS was added to the cells and left to incubate overnight at 37 °C. 72 h later, the supernatant containing the formed lentivirus was collected from the dish and centrifuged at 3000 rpm for 15 min at 4 °C and filtered using a 0.45 μm filter. 10 mL of the supernatant was added to each of HEK293 and HEK293T at 70% cell confluency in T25 flask for transduction. HEK293 and HEK293T transduced cells expressing the pGreenFire1-NF-κB (EF1α-puro) plasmid were selected for by treating the cells with 2 µg/mL puromycin (Gibco #A1113803) for three weeks. Cells were maintained in DMEM containing 10% FBS and 0.5 µg/mL puromycin.

### Linear ubiquitin binding assay

Transfected HEK293T cells were harvested and lysed with RIPA buffer (Thermo Fisher Scientific #89901) supplemented with protease and phosphatase inhibitor (Thermo Fisher Scientific #1861280). The overexpressed proteins were isolated using the µMACS GFP Isolation Kit (Miltenyi Biotec #130-091-125). The lysates were quantified using Pierce BCA Protein Assay Kit (Thermo Fisher Scientific #23225) and 1 mg of each was loaded onto the columns and washed 5 times with IP reaction buffer (50 mM Tris-HCl pH 8.0, 150 mM NaCl, 1 mM DTT, 0.1% NP-40). The proteins were then incubated on the column with 0.5 µM M1-linked Tetra-Ubiquitin (South Bay Bio #SBB-UP0119) diluted in IP reaction buffer for 1 h at 4 °C before eluting the columns as per the kit instructions.

### p65 phosphorylation

Transfected HEK293 cells were harvested and stimulated in suspension with 20 ng/mL TNF (Thermo Fisher Scientific #300–01 A) for the indicated times and then washed twice with cold PBS. The cells were lysed in boiling 1% SDS 10 mM Tris-HCl pH 8.0 and then quantified with Pierce BCA Protein Assay Kit prior to western blotting. Alternatively, they were fixed with Lyse/Fix Buffer (BD Biosciences #558049) and permeabilized with Perm Buffer II (BD Biosciences #558052) as per the manufacturer’s instructions and then stained with phospho-NF-κB p65-PE prior to flow cytometric analysis.

### NF-κB-dependent GFP reporter assay

Transfected HEK293-NF-κB-GFP were stimulated with 10 ng/mL TNFα for 24 h. The cells were then harvested, washed with cold PBS, and incubated for 15 min on ice with the LIVE/DEAD Fixable Near-IR Dead Cell Stain (ThermoFisher Scientific #L10119) prior to acquisition via flow cytometry.

### Data acquisition and analysis

All flow cytometry experiments were acquired on Cytek Aurora (Cytek Bioscences) and analyzed via FlowJo software v10.10.0 (BD). Statistical analysis was performed on GraphPad Prism v.10.2.3 (GraphPad Software) using either a two- or one-way ANOVA with Tukey’s multiple comparison test where appropriate. All error bars shown represent the standard error of the mean.

## Results

### A patient with a homozygous recessive missense mutation in A20 ZF7

The patient is a 21-year-old female of Pakistani descent born to consanguineous parents. She was diagnosed with SLE at 4 years of age, and this was associated with severe clinical manifestations such as antiphospholipid syndrome, autoimmune hemolytic anemia, autoimmune thrombocytopenia, autoimmune thyroid disease, irregular bowel motion, chronic diarrhea, and gallbladder stones, as well as recurrent vascular headache. Her serology was positive for antinuclear antibody, anticardiolipin antibody, anti-thyroid peroxidase, and anti-intrinsic factor. The patient was treated with immunosuppressants at the time of recruitment including mycophenolate mofetil, prednisone, and deflazacort. The patient’s condition improved following treatment with prednisone and IV rituximab and was later maintained on azathioprine before being lost to follow-up.

The proband’s 45-year-old mother was diagnosed with SLE at the age of 28 and had suffered milder symptoms such as erythematous skin rash involving face and neck, recurrent oral ulcers, hair loss, intermittent joint pain and swelling, lower back pain, and osteoarthritis. The mother tested positive for antinuclear antibody and anti-double-stranded DNA antibody and had a skin biopsy that showed pathological features highly suggestive of SLE. Initially, the mother was managed with physiotherapy, analgesics, and non-steroidal anti-inflammatory drugs, but was eventually escalated to prednisone and methotrexate five years following recruitment.

**Figure 1:**
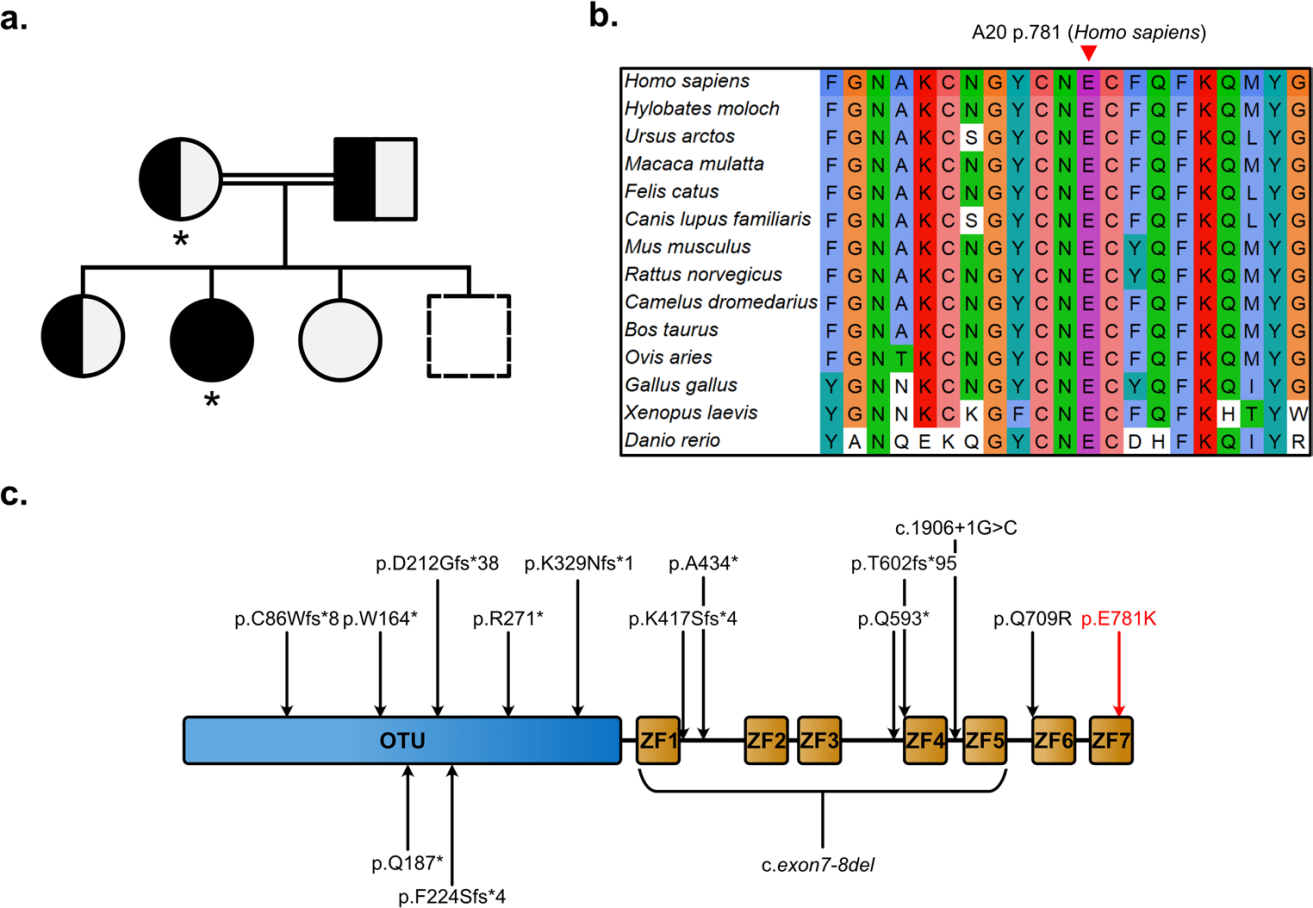
Fig. 1 The single amino acid substitution in A20, E781K, identified in the proband is in the linear ubiquitin binding domain of A20. **a** A pedigree showing the segregation of A20 E781K mutation within the family. Asterisks indicate the homozygous proband and the affected mother. Dashed line was not subjected to whole exome sequencing. **b** A20 ZF7 amino acid conservation across species. **c** Schematic of A20 domains highlighting the positions of previously described HA20 mutations associated with SLE, neuropsychiatric SLE, or lupus nephritis [49]. The position of the mutation in the current study is highlighted in red

**Figure 2:**
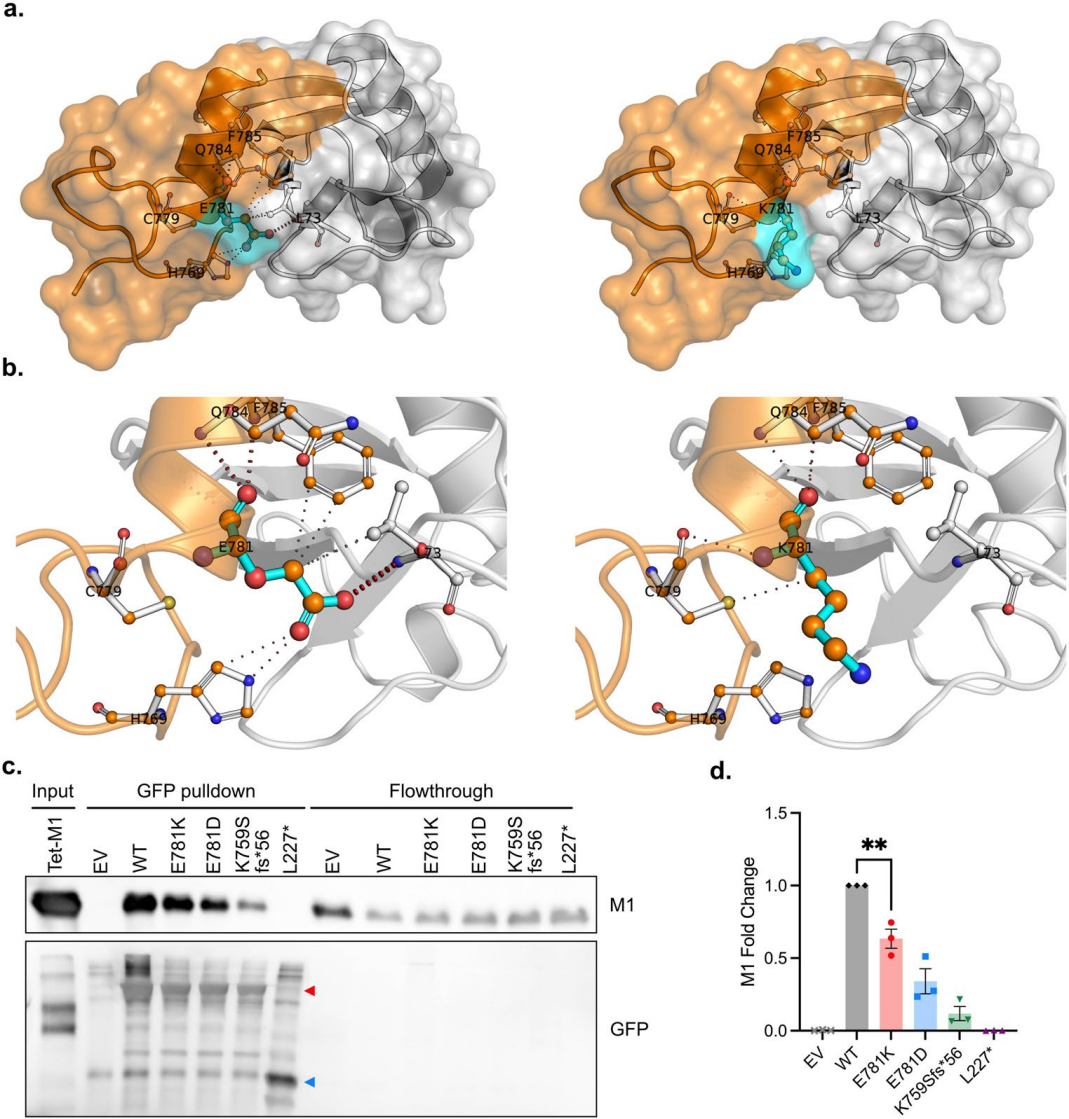
Fig. 2 A20 E781K disrupts binding to linear ubiquitin. **a** DynaMut2 analysis of ZF7-linear ubiquitin complex, showing wild-type (left) and mutant (right) forms, highlighting the disruption to the linear ubiquitin binding site. ZF7 is depicted in orange and linear ubiquitin in light grey. **b** The same DynaMut2 analysis shows disrupted inter- and intramolecular interactions within ZF7 and between ZF7 and linear ubiquitin. Hydrogen bonds are shown in red and hydrophobic interactions in dark grey. Predicted bonds that did not change between wild-type and mutant are not shown for clarity. **c** Pulldown of tetra-linear ubiquitin (M1) by A20-GFP extracted from transfected HEK293 cells. pmaxGFP was used as the empty vector (EV) control. Red arrow indicates the expected molecular weight of the WT and ZF7 mutant A20-GFP bands, blue arrow indicates the expected molecular weight of the L227* A20-GFP truncated mutant. The figure is representative of 3 independent experiments. **d** Western blot quantification showing the fold change of linear ubiquitin binding to A20 mutants compared to WT. Only statistics for WT vs A20 E781K is shown. *P* value was calculated using one-way ANOVA followed by Tukey’s multiple comparisons test. **P < 0.01

Whole-exome sequencing of the proband, the parents, and two healthy siblings revealed a homozygous missense mutation in *TNFAIP3* c.2341G > A corresponding to p.E781K in A20 in the proband. Both parents and one sibling are heterozygous for the mutation, with only the proband and the mother being affected (Fig. 1a). No other mutations in genes associated with SLE were detected. A20 E781 is in the ZF7 domain of A20 and is highly conserved across species (Fig. 1b, c). The variant was predicted to be damaging based on deleteriousness prediction algorithms (Table 1). In silico structural modeling of the ZF7 domain-linear ubiquitin complex reveals that A20 E781K significantly alters ZF7’s linear ubiquitin binding site, which could affect its nonenzymatic capability to bind linear ubiquitin (Fig. 2a, b). This is further supported by the fact that DynaMut2 analysis predicted destabilization of the complex as evidenced by the negative change in Gibbs free energy (Table 1) [28].

This A20 E781K variant is extremely rare with an allele frequency of 1.415 × 10^− 6^, present in gnomAD v4.1.0 in two individuals of Southeast Asian descent and no reported homozygotes. Recently, this variant has been reported on ClinVar as a variant of unknown significance (VUS) with supporting computational evidence (PP3). No protein loss-of-function variants have been reported at this position, and no homozygous loss-of-function germline variants have been reported anywhere in this gene. Other rare homozygous variants identified in the proband were excluded based on their lack of relevance to the patient’s clinical phenotype (Supplementary Table 1).

**Table 1:** Summary of in silico predictions of A20 E781K stability and pathogenicity

Algorithm	Score	Prediction
SIFT	0.01	Damaging
PolyPhen	0.999	Probably damaging
Mutation taster	–	Disease causing
CADD Phred	33	–
AlphaMissense	0.968	Pathogenic
DynaMut2	− 0.86 kcal/mol	Destabilizing

### A20 E781K fails to bind linear ubiquitin in vitro

Since A20 E781K was predicted to disrupt the linear ubiquitin binding site of ZF7, we investigated the effect of the mutation on A20’s ability to bind linear ubiquitin. Either GFP-tagged wild-type A20 or A20 E781K were overexpressed in HEK293T cells. The mutation A20 E781D, a somatic mutation previously shown to be associated with B cell lymphoma and to disrupt A20 binding to linear tetraubiquitin, as well as A20 K759Sfs*56, which disrupts the entire ZF7 domain, and the early termination A20 L227* mutation were also included as comparator variants with known deleterious effects on A20 function [9, 19, 29, 30]. The A20 proteins were isolated by GFP pulldown and incubated with M1-linked tetra-ubiquitin. While wild-type A20 was readily able to pulldown linear tetra-ubiquitin, A20 E781K exhibited a significant reduction (36.6 ± 6.59%) compared to wild-type A20 in its ability to bind and pulldown linear tetra-ubiquitin. Notably, tetra-ubiquitin bound progressively less to A20 E781D, A20 K759Sfs*56, and L227* (65.9 ± 8.6%, 88.17 ± 4.9%, 99.8 ± 0.1%, respectively), consistent with their increasing disruptive impact on A20 structure and underscoring the important role of the ZF7 domain in linear ubiquitin binding (Fig. 2c, d, Supplementary Table 2). This was accompanied by a reciprocal trend in the flowthrough fraction, with efficient depletion of linear tetra-ubiquitin by A20 wild-type, whereas increasing amounts of tetra-ubiquitin remained in the flowthrough for the mutants in proportion to their structural disruption of A20 (Fig. 2c).

### A20 E781K confers enhanced NF-κB activation

**Figure 3:**
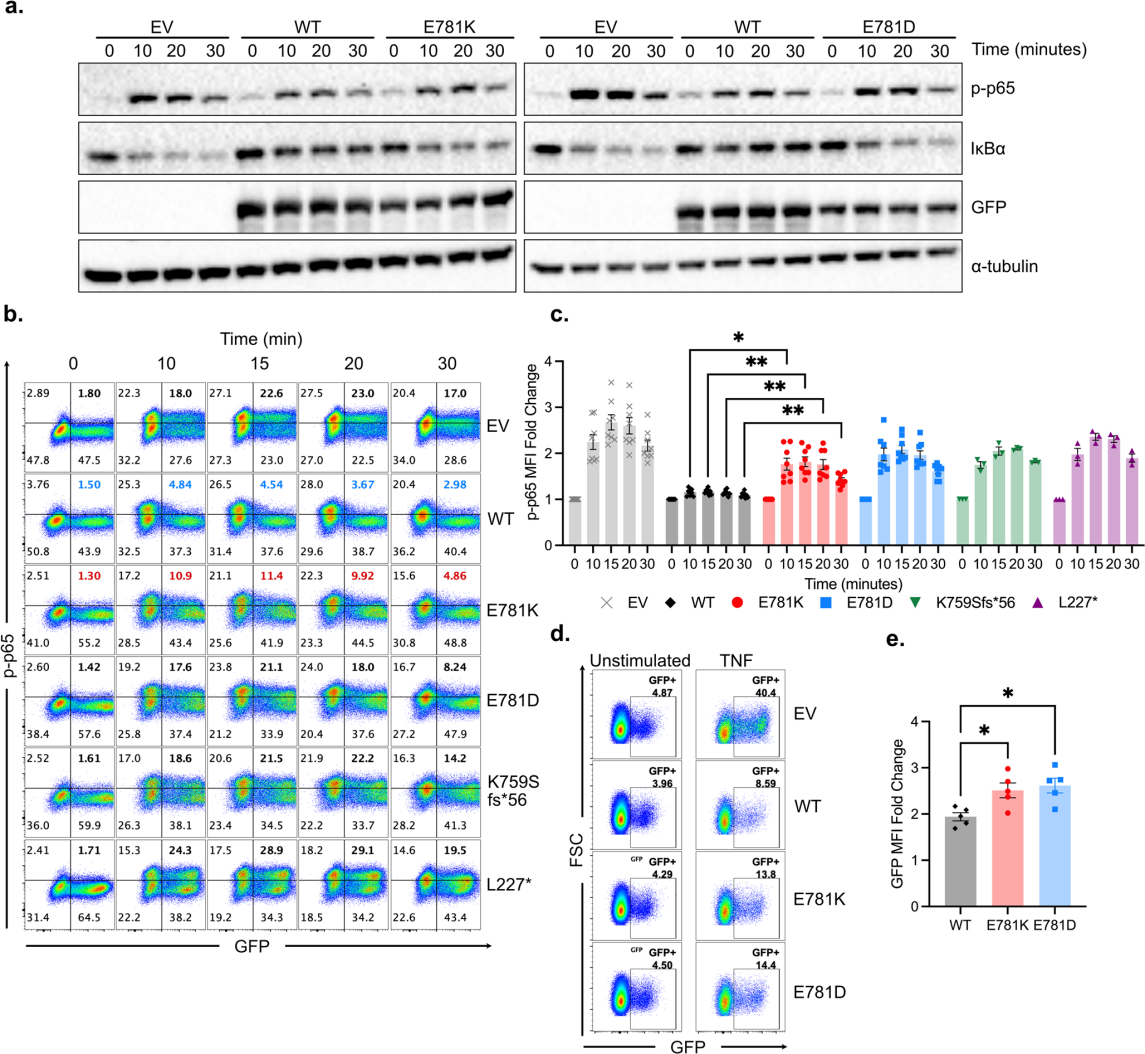
Fig. 3 Enhanced NF-κB activation and downstream signaling with A20 E781K compared to WT. **a** HEK293 cells were transfected with either pmaxGFP (EV) or A20-GFP constructs and stimulated with 20 ng/mL TNF for the indicated times. **b** Flow cytometric detection of p-p65 levels in A20-GFP-transfected HEK293 following stimulation with 20 ng/mL TNF for the indicated times. The figure is representative of 8 independent experiments, with the last three experiments including A20 K759Sfs*56 and A20 L227*. P value was determined using a two-factor mixed-effects model followed by Tukey’s multiple comparisons test. **c** Fold change increase in mean fluorescence intensity of p-p65 in GFP+ cells over time relative to the unstimulated condition. Only significance between WT and A20 E781K is shown. P value was calculated using two-way ANOVA followed by Tukey’s multiple comparisons test. **d** NF-κB reporter assay showing the increase of GFP+ population in pcDNA3-mRFP (EV) or A20-RFP transfected cells following stimulation with 10 ng/mL TNF for 24 hours. GFP fluorescence intensity was measured in RFP+ cells. The figure is representative of 5 independent experiments. **e** Fold change in the mean fluorescence intensity of GFP in the RFP+ population relative to the unstimulated condition. *P* value was calculated using one-way ANOVA followed by Tukey’s multiple comparisons test. *P < 0.05, **P <0.01

To determine if the reduced ability of A20 E781K to bind linear ubiquitin compromises its ability to suppress NF-κB activation akin to that of A20 E781D, the GFP-tagged constructs were overexpressed in HEK293 cells and stimulated with TNF over a period of 30 min. NF-κB activation was assessed by detecting the phosphorylation of NF-κB p65 subunit as well as the degradation of IκBα via western blot. Both A20 E781K and E781D mutations exhibited enhanced p65 phosphorylation and IκBα degradation compared to wild-type A20 as early as 10 min following stimulation, suggesting a reduced capacity to regulate the NF-κB pathway (Fig. 3a). Flow cytometry was used to assess and quantify degree of p65 phosphorylation in A20-GFP+ cells. Both A20 E781K and E781D transfected cells demonstrated a significant increase of phospho-p65 upon stimulation at all timepoints, peaking at 1.82 ± 0.12-fold, and 2.08 ± 0.11-fold increase, respectively, 15 min following stimulation compared to unstimulated cells. Analysis of previously published mutations A20 K759Sfs*56 and A20 L227* yielded even higher phosphorylation of p65 reaching a maximum of 2.09 ± 0.01 and 2.36 ± 0.06-fold change at 20 and 15 min following stimulation, respectively. Notably, neither A20 K759Sfs*56 nor A20 L227* was distinguishable from the empty vector control in their effects on p65 phosphorylation at any time point, consistent with loss of function. Wild-type A20-transfected cells, on the other hand, induced p65 phosphorylation only marginally, peaking at 1.17-fold increase by 15 min (Fig. 3b, c, Supplementary Table 3). This assay revealed a distinct effect of the wild-type A20 effectively suppressing p65 phosphorylation, whereas all mutants showed enhanced phosphorylation compared to wild-type. Meanwhile, in GFP- cells, p65 was readily phosphorylated upon stimulation with TNF across all conditions (Fig. 3b). By 30 min, p65 phosphorylation was still significantly elevated in the mutants compared to wild-type A20, suggesting prolonged NF-κB activation in the absence of wild-type A20 (Fig. 3b, c, Supplementary Table 3). Overall, these results confirm that A20 E781K causes an enhanced activation of the NF-κB pathway.

To investigate if A20 E781K enhances the transcription of NF-κB downstream target genes, we constructed the HEK293-NF-κB-GFP reporter cell line that expresses GFP upon NF-κB activation. The reporter cells were transfected with the RFP-tagged A20 constructs and stimulated with TNF overnight. Flow cytometric analysis revealed significantly enhanced production of GFP in reporter cells transfected with A20 E781K and A20 E781D compared to wild-type transfected cells. Specifically, GFP fluorescence intensity increased by 2.51 ± 0.16-fold and 2.62 ± 0.16-fold, respectively, relative to unstimulated cells, whereas wild-type A20 transfected cells only showed a 1.94 ± 0.09-fold increase in GFP production (Fig. 3d, e). Taken together, these results show the requirement for a functional A20 ZF7 to effectively abrogate NF-κB downstream target gene expression.

## Discussion

While A20 was first identified in the early 1990s as a molecule that protects human endothelial cells from TNF induced apoptosis, it emerged as a key anti-inflammatory molecule through its function within the NF-κB pathway [31]. Its involvement in human pathology is still being investigated as mutations in A20 result in a wide range of manifestations and are of varied penetrance even within members of the same family [20]. Yet, the fact that all known human A20 mutations are heterozygous indicates that at least one functional copy is required for survival. This is consistent with the mouse models in which complete inactivation of A20 results in perinatal lethality, while mice haploinsufficient for A20 develop neuroinflammation that evolves to progressive neuropsychiatric SLE [21, 32]. Moreover, tissue-specific knockout mice develop manifestations reminiscent to those of HA20 depending on the knockout cell type such as joint damage, SLE, or autoantibodies, to name a few [22, 33, 34]. Given that A20 E781K is towards the C-terminal of the protein, it is likely that the intact functions of the OTU and ZF4 domains combined with the fact that ZF7 was able to retain some of its linear ubiquitin binding ability allowed for the A20 E781K homozygous patient to thrive. Similarly, it was shown that mice with specific inactivation of ZF7 are born at Mendelian ratios and do not die after birth, but exhibit progressive multiorgan autoinflammatory disorder, indicating that functional OTU and ZF4 domains are sufficient to rescue the perinatal lethality [23, 24].

A20 E781K mutation exhibits impaired binding to linear ubiquitin compared to wild-type (Fig. 2c). It was previously shown that A20 ZF7 binds linear diubiquitin moieties through a series of hydrogen bonds and hydrophobic interactions [9]. Residue E781 is critical to binding linear ubiquitin, as it forms a hydrogen bond with D32 of the proximal linear ubiquitin moiety via its backbone amide group. It also forms hydrogen bonds with L73 on the distal linear diubiquitin moiety and H769 of ZF7 via its side chain. DynaMut2 analysis of the E781K substitution indicates that the introduced lysine points away from the linear ubiquitin binding site. Due to the nature of the lysine side chain and its structure, it is unable to form a hydrogen bond with L73 of the distal linear ubiquitin moiety. The mutation is also predicted to disrupt interatomic interactions within ZF7, specifically the loss of hydrogen bonding between K781 and H769, which could result in the predicted instability of the domain (Fig. 2a, b; Table 1). Additionally, A20 E781 and F785 along with L73 of the distal linear ubiquitin moiety are part of the hydrophobic surface that interacts with the linear ubiquitin [9]. Structural prediction shows E781 forms hydrophobic interactions with both A20 F785 and L73 of the distal linear ubiquitin moiety, resulting in a “pocket”-like structure that allows for the hydrogen bonding of L73 of distal ubiquitin. The introduction of K781 to the structure abolishes these hydrophobic interactions, causing a collapse of this pocket (Fig. 2a, b). These predictions could shed light on how the E781K substitution affects ZF7’s ability to bind linear ubiquitin.

To investigate this, we directly compared wild-type A20’s ability to bind linear tetra-ubiquitin to that of A20 E781K along with a panel of previously published mutants in vitro. Wild-type A20 readily bound linear ubiquitin while A20 E781K exhibited a marked reduction in its binding ability. Disruption of the ZF7 domain in A20 K759Sfs*56 resulted in only minimal binding. Both the truncating A20 L227* variant and the empty vector failed to bind linear ubiquitin (Fig. 2c, d, Supplementary Table 2). These results suggest that while the ZF4 domain may have some capacity to independently bind linear ubiquitin, optimal binding depends on having an intact ZF7 domain. Interestingly, while A20 E781K and E781D are both single residue substitutions at this same site, a lysine substitution appears to have less of an impact on binding to linear ubiquitin than an aspartate substitution (Fig. 2c, d, Supplementary Table 2). Despite maintaining the negative charge in A20 E781D, the longer and more flexible lysine side chain of A20 E781K may provide a more favorable structural geometry to retain some binding to linear ubiquitin.

The direct consequences of this disrupted binding are reflected in increased phosphorylation of p65, increased degradation of IκBα, and increased NF-κB transcriptional activity compared to wild-type A20 as evidenced by the NF-κB reporter assay (Fig. 3a–e). While the baseline levels of NF-κB activation between E781K and wild-type are similar, phosphorylation of p65 does not return to the same levels as those of wild-type following stimulation with TNF, indicating a delayed shutdown of the pathway with mutant A20 (Fig. 3a, c). Even though HEK293 cells are a valid model to investigate NF-κB activity, immune cells can express a much greater level of NF-κB and LUBAC components, which could amplify the effect of the mutation in patient cells [35]. Translated to the patient, these findings suggest that the clinical manifestations observed in the patient – and to a less extent, her mother – likely result from excessive NF-κB activation and impaired suppression of the pathway, further highlighting the critical role of A20 ZF7 in maintaining immune homeostasis.

The case at hand provides valuable insight into the clinical heterogeneity of A20-associated pathologies. The homozygous proband carrying the A20 E781K variant developed a more severe phenotype than the heterozygous mother, underscoring the requirement for a functional ZF7 domain. This is evidenced by the fact that while both have SLE as a shared manifestation, the patient was diagnosed at 4 years of age, whereas the mother was diagnosed at 28 years of age. The number of functional copies of A20 ZF7 may influence disease severity and penetrance. While the proband exhibited a more severe systemic autoimmunity with hematological involvement, manifesting as autoimmune hemolytic anemia and autoimmune hypothyroidism, the mother presented with a more classical SLE phenotype with musculoskeletal and mucocutaneous involvement. Moreover, the patient has a healthy 22-year-old sister who is heterozygous for the A20 variant but exhibited no overt symptoms at the time of recruitment. This observation may suggest incomplete penetrance of disease or a delayed onset of symptoms for A20 E781K heterozygous individuals. Previous studies suggest that HA20 mutations can result in heterogenous clinical expression, even among members of the same family. The patient’s father, who is also heterozygous for the A20 E781K mutation, is reportedly unaffected. Given that the E781K mutation appears to be hypomorphic and less disruptive than other HA20-associated loss-of-function mutations, we speculate that this may contribute to the reduced disease penetrance observed in heterozygous carriers.

The variable disease expression observed in this family is consistent with the increasingly recognized heterogeneity of inheritance models in systemic autoimmune/ autoinflammatory disorders (SAIDs), in which penetrance, hypomorphic effects, and gene dosage collectively influence both inheritance patterns and clinical phenotypes. Genes such RIPK1, OTULIN, CARD11, NLRP1, and MEFV/Pyrin have been found to follow mixed inheritance models depending on the functional consequences of specific variants [36–44]. While some such as STING1 and NLRC4 are associated with a dominant mode of inheritance, these may be redefined in the future as variant-specific inheritance patterns are identified [45, 46]. Hypomorphic variants may be tolerated in the heterozygous state and manifest disease only when inherited recessively. For example, a recently-described hypomorphic variant in CTLA4 was reported to follow a recessive mode of inheritance, challenging the classical autosomal dominant model typically associated with CTLA4 insufficiency [47, 48]. In this case, we describe a hypomorphic A20 mutation in which clinical manifestation appears to be influenced by gene dosage and penetrance, in contrast to the typical autosomal dominant inheritance observed for pathogenic A20 loss-of-function-variants.

When comparing this mutation with previously reported mutations in HA20 patients, we identified one report of a family with three generations carrying the heterozygous frameshift mutation p.K759Sfs*56 affecting only the ZF7 domain [30]. The proband of that case developed an autoinflammatory phenotype manifesting in hematochezia, steatorrhea, diarrhea, recurrent fever spikes, skin rashes, as well as oral ulcers following viral infections that eventually resolved. Members of that family with the same mutation also developed oral and genital ulcers, diarrhea, and had a history of Behçet’s disease diagnosis. While there is clinical similarity between HA20 patients from this case and our proband’s mother, particularly the oral ulcers and skin rashes, the A20 K759Sfs*56 heterozygous mutation appeared to result in greater disease penetrance than our A20 E781K mutation. This could be attributed to the frameshift mutation more severely abrogating A20’s ZF7 domain function, while the A20 E781K mutation showed some residual function as demonstrated by the linear ubiquitin binding assay (Fig. 2c, d, Supplementary Table 2). NF-κB activation was also higher with A20 K759Sfs*56 than with A20 E781K (Fig. 3b, c, Supplementary Table 3), in line with the greater clinical penetrance of the frameshift variant. However, the fact that HA20 mutations vary greatly in their clinical manifestations could also explain the heterogeneity of HA20 phenotype exhibited across patients [20].

It was recently reported in a systematic review of 191 HA20 patients that only 16 of them developed SLE [49]. Consistent with the heterogeneity of HA20 manifestations, these patients all had varied clinical expression, with mucocutaneous and musculoskeletal involvement being the most common, while some also had hematological manifestations. The age of SLE diagnosis of these patients also varied greatly ranging from 1 to 34 years of age. This range of diagnoses correlates with the age of onset and may be more indicative of disease penetrance rather than severity in the context of HA20 patients. However, in our case, the proband’s autosomal recessive A20 E781K mutation led to earlier and more severe clinical manifestations compared to the heterozygous mutation carried by the mother. The reported mutations associated with SLE in these patients were not concentrated in a specific region of the gene and typically affected one or more domains via frameshifts, premature stop codons, or exon deletions (Fig. 1c) [49]. Notably, none of these mutations specifically targeted the ZF7 domain. Given that our proband’s mother is haploinsufficient for A20 ZF7, we propose that defects in this ZF7 domain may also contribute to the development of lupus-like symptoms in HA20 patients.

Overall, in this study, we identified a patient carrying a homozygous substitution in the A20 ZF7 domain with an affected heterozygous mother. Both proband and mother have SLE, but the proband had disease onset in early childhood with more severe autoimmune manifestations. The family was lost to follow-up, and as a result, patient-derived cells were unavailable for analysis, representing a significant limitation of the study. To investigate the functional impact of the variant, structural modeling and overexpression systems were used to demonstrate that it impairs A20 binding to linear ubiquitin. This disruption resulted in hyperactivation and defective resolution of the NF-κB pathway, consistent with increased transcription and translation of NF-κB downstream target genes. These findings may explain the patient’s phenotype, as hyperactivation of the NF-κB pathway leads to several autoimmune and autoinflammatory phenotypes as seen in other NF-κB-related pathologies such as in RIPK1, NEMO, OTULIN, and CARD11 deficiencies [36, 39, 40, 50]. This case represents a novel inborn error of immunity, which we term *Insufficiency of A20 (IA20)*, as it is the first documented case of autosomal recessive A20 disease, resulting in an early-onset lupus-like disorder with systemic autoimmunity.

## Supplementary Information

Below is the link to the electronic supplementary material.

Supplementary Material 1.

## Data Availability

The source data presented in this article can be made available upon request to the corresponding author.
