# Supplementary material for: Autosomal recessive A20 zinc finger 7 mutation is associated with early-onset lupus-like disease
Source: Inflamm Res. 2026 Jul 18;75(1):180. doi: 10.1007/s00011-026-02326-2 (PMC13380576; doi:10.1007/s00011-026-02326-2)
Supplement: Supplementary file 1 — Supplementary Material 1. [file 11_2026_2326_MOESM1_ESM.pdf]

Supplementary Table 1: Rare homozygous variants identified in the proband

Gene	Chromosomal Position	Coding Sequence Change	Protein Change	Poly Phen	SIFT	Mutation Taster	CADD Phred
TNFAIP3	chr6:138202424 G>A	c.2341G>A ENST00000237289.4	p.E781K	PrD	D	DC	33
LRRC8E	chr19:7963672 G>A	c.265G>A ENST00000306708.6	p.G89S	PrD	T	DC	24.5
STXBP2	chr19:7707343 C>T	c.823C>T ENST00000221283.5	p.R275W	PrD	D	Poly	22.5
PDE4A	chr19:10559813 G>A	c.607G>A ENST00000352831.6	p.V203I	B	T	Poly	12.32
AP1M2	chr19:10697883 G>T	c.25C>A ENST00000250244.6	p.L9M	PD	T	DC	25.1
SEPT4	chr17:56606533 C>T	c.3G>A ENST00000317268.3	p.M1?	B	D	DC	19.07
S1PR2	chr19:10335446 C>T	c.136G>A ENST00000590320.1	p.A46T	B	D	DC	19.15

PolyPhen scores: B = Benign, PD = Possibly Damaging, PrD = Probably Damaging
SIFT scores: T = Tolerated, D = Damaging
Mutation Taster scores: Poly = Polymorphism, D = Disease Causing

Supplementary Table 2: Summary of Tukey's multiple-comparisons test for the pairwise comparisons shown in Fig. 2d

Comparison	
EV vs. WT	***
EV vs. E781K	***
EV vs. E781D	**
EV vs. K759Sfs*56	ns
EV vs. L227*	ns
WT vs. E781K	**
WT vs. E781D	***
WT vs. K759Sfs*56	***
WT vs. L227*	***
E781K vs. E781D	*
E781K vs. K759Sfs*56	***
E781K vs. L227*	***
E781D vs. K759Sfs*56	ns
E781D vs. L227*	**
K759Sfs*56 vs. L227*	ns

ns (not significant), $P \geq 0.05$, *$P < 0.05$, **$P < 0.01$, ***$P < 0.001$

Supplementary Table 3: Summary of Tukey's multiple-comparisons test for the pairwise comparisons shown in Fig. 3c

Comparison	Time (minutes)			
	10	15	20	30
EV vs. WT	**	***	***	***
EV vs. E781K	ns	**	*	**
EV vs. E781D	ns	ns	ns	*
EV vs. K759Sfs*56	ns	ns	ns	ns
EV vs. L227*	ns	ns	ns	ns
WT vs. E781K	*	**	**	**
WT vs. E781D	**	***	***	***
WT vs. K759Sfs*56	*	*	***	***
WT vs. L227*	ns	**	**	*
E781K vs. E781D	ns	ns	ns	ns
E781K vs. K759Sfs*56	ns	ns	ns	***
E781K vs. L227*	ns	*	*	ns
E781D vs. K759Sfs*56	ns	ns	ns	ns
E781D vs. L227*	ns	ns	ns	ns
K759Sfs*56 vs. L227*	ns	ns	ns	ns

ns (not significant), $P \geq 0.05$, $^{*}P < 0.05$, $^{**}P < 0.01$, $^{***}P < 0.001$
